# Permeation of metallic gunshot residues in a commercial gun cleaning solvent through a disposable nitrile glove

**DOI:** 10.1007/s12024-025-01077-6

**Published:** 2025-09-29

**Authors:** Travis Cribbs, Shane Que Hee

**Affiliations:** Present Address: Department of Environmental Health Sciences, Fielding School of Public Health, University of California Los Angeles, 650 Charles E Young Jr Drive South, Los Angeles, CA 90095-1772 USA

**Keywords:** Gunshot residue sampling and analysis, Gunshot cleaning solvent, Nitrile glove permeation, ICP-MS

## Abstract

*Purpose. *Gunshot residue (GSR) accumulates in firearms and must be removed mechanically and with gun cleaning solvent (GCS). There are no peer review journal publications on what gloves might be resistant to GSR being cleaned with a GCS. The aim was to fill the data gap. *Methods*. A Kimtech Science Blue disposable nitrile glove was challenged in triplicate by dissolved GSR of diameters less than 125 micrometers in a popular GCS with permeates collected in n-decane at 35 °C in a ASTM F739-20 glass permeation cell with no collection side recirculation. Samples from the collection and challenger sides and permeated glove pieces were analyzed for metals by solvent evaporation, concentrated acid digestion and then inductively coupled plasma-mass spectrometry. *Results*. Lead, copper and barium GSR compounds permeated between 240 min and 480 min, also being detected within the permeated glove material. The GSR concentrations in mg/g were lead 86.8 ± 0.2, copper 96.4 ± 0.2, and barium 0.622 ± 0.012. *Conclusion*. A practical and inexpensive method to resist the toxic elements in GSR in a popular GCS for at least 4 hours was to wear a disposable Kimtech Science Blue nitrile glove of average thickness 143-148 µm. More gloves, GCS and GSR should be tested.

## Introduction

Forensics personnel and investigators often clean guns of gunshot residue (GSR or fouling) after or during criminal investigations and trials. The GSR is removed by brushing and applying gun cleaning solvent (GCS). Yet there are no published peer review science journal articles on what glove types should be worn for protection. A 2024 article has appeared on permeation of a GCS of over 250 components through disposable nitrile gloves [1]. Wearing gloves is the major personal protective equipment (PPE) for protection from hand skin sensitization, dermatitis and adverse systemic effects after dermal exposure and absorption of chemicals [2, 3].

GSR consists of many complex inorganic, organic and organometallic components [4–6]. GSR can be detected in the environment after gun firing but also inside the gun itself from where it needs to be removed to prevent malfunctions like jamming, misfires, and decreased accuracy from factors like internal corrosion, rusting and GSR bullet obstruction and misalignment. Most of the GSR studies have focused on the inorganic elements and the influence of cleaning efficiency relative to the gun “memory effect” in comparing environmental GSR with the GSR within a suspect’s gun [7, 8].

The present aim was to fill the data gap by testing the hypothesis that commercial firearm solvents facilitate the permeation through disposable nitrile glove material of dissolved metallic GSR compounds produced during the operation of the firearm. This involved generating the GSR from a common firearm and cartridge, characterizing the GSR relative to particle size, defining a GSR solvent based on commercial recommendations, performing the permeation test with a modified ASTM F739 protocol, and quantifying the metals by digestion and inductively coupled plasma-mass spectrometry (ICP-MS).

## Materials and methods

### GSR generation

GSR was initially generated with a Ruger Mini-14 rifle chambered in 5.56 × 45 mm NATO (Big 5 Sporting Goods, Santa Paula, CA) by firing 1000 rounds of Federal 5.56 × 45 mm NATO 55gr Full Metal Jacket ammunition (Turner’s Outdoorsman. Oxnard, CA) at a firing rate of approximately 10 rounds per minute. The GSR generation occurred between 10am and 12pm at an outdoor location in southern California chosen for its seclusion and lack of animal and plant life. The weather was clear and without wind. All brass ejected from the firearm during the GSR generation was collected and accounted for. The GSR generated was removed from the firearm receiver and bolt with a nylon cleaning brush and removed from the barrel with a nylon bore brush. The GSR was collected into a pre-weighed Teflon vial and capped. The vial was placed into a cooler with ice packs and stored there until transported to the laboratory, where it was finally stored in a −20 ± 2 °C freezer until analysis. The GSR handler wore Kimberly Clark’s Kimtech Blue disposable gloves that were powderless, unsupported and unlined (Fisher Scientific, Chino, CA), the glove tested for GSR permeation.

### GSR particle sizing

The collected GSR container was weighed (Mettler AE260 Analytical Balance, Hightstown, NJ) to measure the total mass of the collected GSR. The GSR was passed through a three-stage sieve column with sieve opening sizes of 420, 177, and 125 μm (Fisher Scientific, Chino, CA). All sieves and the final collection cup were weighed.

### GSR solvent

The magazine *Outdoor Life* has annually classified Hoppe’s No. 9 Gun Bore Cleaner as the best overall GCS based on its ability to remove primer, powder, and fouling (metal and organic), and to prevent rust. The Safety Data Sheet [9] indicated a non-polar fraction of kerosene (30–60%) plus acetate esters (2–10%) and a polar alcoholic fraction with ethyl alcohol dominant (10–30%), isopropyl alcohol (5–10%), and methyl alcohol (1–5%). The gas chromatograph-mass spectrometer (GC-MS) characterization [1] revealed that the ethyl alcohol content was 29.7% ± 5.9% or about 30%. There were at least 250 distinct peaks in the chromatogram.

### GSR permeation

A mass of 5.0 mg of < 125 μm GSR was dissolved in 30 mL Hoppe’s no. 9 Gun Bore Cleaner GCS as a challenge solution for permeation testing. The palms of four Kimberly Clark Kimtech Science Blue Nitrile gloves (Fisher Scientific, Chino, CA) were cut into 45 mm diameter circular pieces, inspected for holes (American Optical MicroStar hand-held microscope, Buffalo, NY), and then measured for thickness (Electronic Digital Micrometer Model CO-030025 with 0-25 mm, 0.001 resolution) and weight (Mettler AE260 Analytical Balance, Hightstown, NJ). The glove pieces were assembled into four ASTM F739 [10] compliant Pyrex permeation test cells, each with a 1-inch (25.4 mm) internal diameter opening between cells (Model I-PTC-600, Pesce Lab, Kennett Square, PA). This cell is equivalent to that of the ASTM D6978-05 [11] for chemotherapy drug permeation since it is the same as the large version of the ASTM F739 cell. Deionized water was introduced to one side of the test cell to test for leaks. The cell was then emptied and disassembled, and the glove pieces were placed into a desiccator at 55 ± 1% relative humidity (a saturated aqueous mixture of sodium dichromate (99%) from Fisher Scientific, Chino) and a temperature of 27 ± 1 °C for 24 h prior to permeation testing. The collection fluid, 10 mL of n-decane (Fisher Scientific, Chino), was added to all cells. n-Decane (that simulates the non-polar kerosene fraction of the GCS) did not degrade or back-permeate Kimtech Blue nitrile [1]. The shorter chain solvent octane was known not to permeate the thinner Sterling nitrile disposable glove within 480 min compared with heptane 134 min, jet fuel (kerosene) 82 min, and hexane 16 min [12]. A volume of 10 mL of the GSR/GCS solution was then introduced into the challenge chamber of three testing cells, and 10 mL of GCS with no GSR was introduced into the challenge cell of a fourth testing cell to be used as a method blank. A modified ASTM F739-20 permeation test was then performed using a water bath shaking at 75 ± 2 rpm and heated to 35 ± 1 °C as recommended in the ASTM D6978-05 protocol [11]. Volumes of 100 µL samples were taken at 0, 5, 10, 15, 20, 30, 40, 60, 120, 240 min after stopping the shaker, and placed into 1-mL Teflon vials. The collection and challenge fluids from each cell were collected at 480 min as well as the dried reconditioned glove pieces and placed into 10 mL Teflon vials.

### Digestions

Digestion of weighed conditioned and reconditioned glove materials was performed with 10 mL volumes each of the concentrated acids, hydrofluoric acid, sulfuric acid, and nitric acid in 100 mL PTFE Teflon beakers on a hot plate (all from Fisher Scientific, Chino, CA) in a fume hood. After initial addition at room temperature, any fumes or reaction were allowed to subside, the Teflon watch glasses replaced, and the temperature ramped to 50 °C for 30 min, 100 °C for 30 min, and refluxing at 150 °C until no red-brown fumes were evolved and no solid residue remained. The Teflon watch glasses were removed to allow liquid evaporation at 90 °C. Calcium D-gluconate solution (10% w/v) was on hand for possible hydrofluoric acid burns and 10% sodium bicarbonate solution for acid spills.

Digestion of permeation collection solutions was similarly performed with 10 mL sulfuric acid and 10 mL nitric acid after evaporation of the permeation cell collection solution at 90 °C.

All evaporated samples after concentrated acid digestions were reconstituted in 10.0 mL 5.0% (v/v) nitric acid, heated for 5 min with Teflon watch glasses on at 90 °C and cooled to room temperature while leaving the watch glasses on before liquid transfer to 15 mL centrifuge tubes with polyethylene one-piece droppers (Fisher Scientific, Chino, CA). Volumes were adjusted to 10.0 mL if necessary.

### ICP-MS analysis

ICP-MS analysis was performed using an Agilent 7500c with octopole collision cell in tandem with an autosampler, the Teledyne Cetac Technologies ASX-560.

The MS ions used for metals of interest were: potassium K (mass/charge m/z 39), nickel Ni (m/z 60), copper Cu (m/z 65), zinc Zn (m/z 60), tin Sn (m/z 120), antimony Sb (m/z 121), barium Ba (m/z 121), and lead Pb (m/z 224). They were quantified using the method of internal standards employing 100 ppb elements in 5%(v/v) nitric acid, with scandium Sc (m/z 45) for K, Ni. Cu and Zn; indium In (m/z 115) for Sn; and bismuth Bi (m/z 209) for Pb.

Standard concentrations for the above combined elements were: 0, 1, 5, 10, 50, 100, 250, 500, 1000, 5000, 10,000, and 100,000 ppb using the appropriate dilutions of the standards concentrates (1000 ppm SPEX CertiPrep obtained from Fisher Scientific, Chino, CA).

The collision cell gases were helium (0.8 mL/min), and hydrogen (1.0 mL/min) with their optimized flow rates set during the tuning step with a tuning solution of 5%(v/v) nitric acid containing 10 ppb each of lithium Li (m/z 7), cobalt Co (m/z 59), yttrium Y (m/z 89), cerium Ce (m/z 140), and thallium Tl (m/z 205) operated at optimized settings to minimize interferences from metal oxides (m/z 156 divided by m/z 140) and doubly charged ions (m/z 70 divided by m/z 140). The constant settings for all three gases were: radiofrequency power, 1430 W; radiofrequency matching, 1.7 V; sample depth, 9.0 mm; torch-horizontal, −1.4 mm; torch-vertical, 0.2 mm; carrier gas flow, 1.5 L/min; nebulizer pump, 0.1 r/s; Peltier spray chamber temperature, 2 °C; Ion Lens (IL) extract, 3.4 V; IL einzel 1, −80 V; IL einzel 2, 14 V; IL cell entrance, −13 V; IL cell exit, −6 V; IL plate bias, −35 V; octopole radiofrequency, 190 V; Quadrupole (Q) atomic mass unit gain, 124; Q atomic mass unit offset, 128; Q axis gain, 0.9993; Q axis offset, 0.06; Q bias, −3.5 V; detector (D) discriminator, 8 mV; D analog high voltage, 1950 V; and D pulse high voltage, 1279 V. The octopole bias was − 7.6 V for hydrogen and argon, with − 5.0 V for helium. The sampling period was 1.2300 s and the signal integration time was 0.1000 s. The cooling water for the torch was at a temperature of 20 °C. The vacua during analyses in pump pascal (Pa) were: backup, 295; annular, 1.99 × 10^−4^.

Automatic sampling occurred in the following order: (1) standards, (2) 10 samples, (3) QA/QC samples of 0, 1,10,100, 0 ppb, with steps 2 and 3 repeated until all samples and any appropriate dilutions are completed, (4) standards. After each sample and standard were read, probe cleaning with 5% nitric acid and water followed also under autosampler control.

After the samples and final standards set were run, the linear regions of the metal standard concentrations were ascertained and the GSR elemental concentrations in the solutions determined by using the regression equations of the linear regions. The actual elemental concentrations in the original GSR were obtained by multiplying the 5.0% nitric acid elemental concentration by the centrifuge tube liquid volume in mL, and dividing by the original GSR weight used to create the 10 mL challenge solution, here 1.67 mg.

## Results and discussion

### Glove and GSR physical properties

The total mass recovered in the particle size GSR results of Table 1 was 15.7 mg dominated by the fine fraction < 125 μm sieve diameter termed the “clean room fraction”, comprising about 64% of the mass from which 5.0 mg was originally weighed for testing for metals.

**Table 1:**
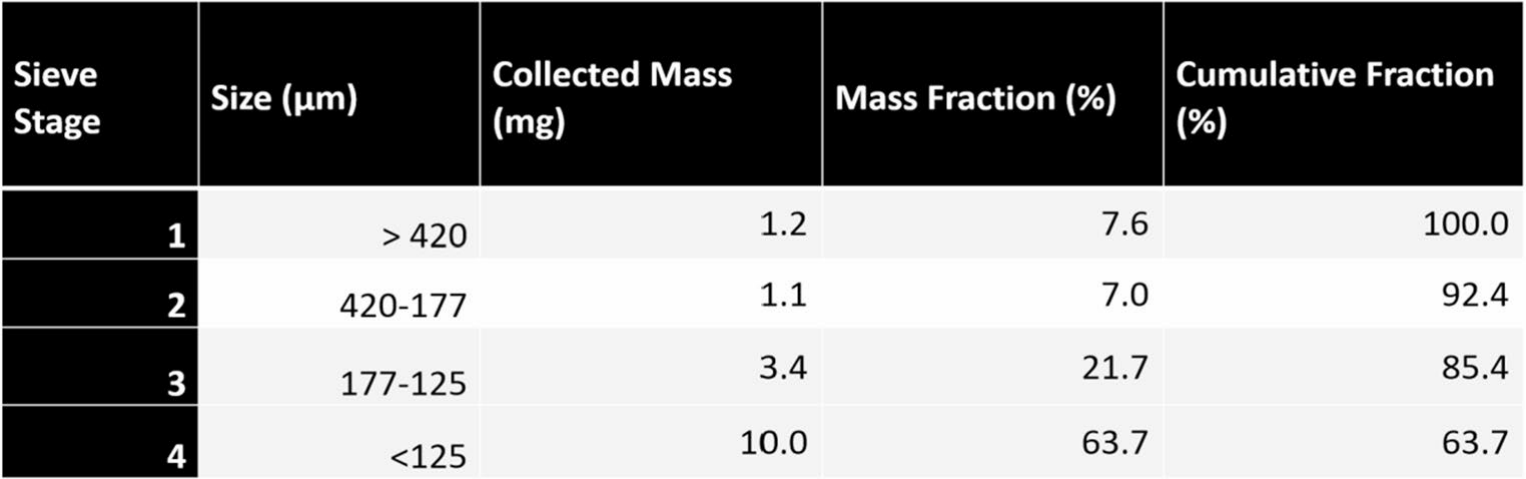
Collected GSR parameters

Table 2 presents the glove piece thickness and weight before and reconditioned after permeation and their differences. Statistically significant intrarun swelling and mass increases at *Student t p* ≤ 0.05 occurred with swelling ranging from 2 to 3% and weight increases ranging from 4.2 to 4.4%. In contrast, the interrun swelling was 4 ± 7% and the interrun weight increase was 4.3 ± 11.8%, both not statistically significant at *p* ≤ 0.05. A swelling classification for individual gloves is available for reference [13]: none (not statistically significant), moderate (about 4%) and complete (10–20%). When there is no swelling, Fick’s first law of diffusion can be used to interpret the permeation data and to calculate diffusion coefficients [2, 3]. The latter is not possible with the present data since a SSPR period was never demonstrated.

**Table 2:** Intrarun and interrun disposable nitrile glove thickness and weight before and after permeation. A: Thickness before (µm); B: Thickness after (µm); C: Thickness difference (µm); D: Thickness difference (%); E: Weight before (mg); F: Weight after (mg); G: Weight difference (mg); H: Weight difference (%)

Glove	A	B	C	D	E	F	G	H
1	148 ± 1	153 ± 1	5 ± 2	3 ± 1	248.0 ± 0.2	258.2 ± 0.2	10.2 ± 0.4	4.1 ± 0.2
2	143 ± 1	147 ± 1	4 ± 2	3 ± 1	251.2 ± 0.1	262.2 ± 0.1	11.0 ± 0.2	4.4 ± 0.1
3	144 ± 1	147 ± 1	3 ± 2	2 ± 1	225.9± 0.1	235.5 ± 0.1	9.6 ± 0.2	4.2 ± 0.1
Average	145 ± 3	149 ± 4	4 ± 7	3 ± 5	241.7 ± 13.8	252.0 ± 14.4	10.3 ± 28.2	4.3 ±11.8

### Glove metals permeation

Table 3 shows the only permeated metals (Pb, Cu and Ba) detected above the ICP-MS lower quantitation limit of 1 ng/mL or 1 ppb (w/v) at 480 min. The permeation began at a time between 240 (when no metals above the blank level were detected) and 480 min. The standardized breakthrough time at the permeation rate of 100 ng/cm^2^/min was not assigned due to the low concentrations of those residues on the challenge side of the cell and the long time interval instead of the mandated 5 min time interval of the ASTM 739 − 20 standard.

**Table 3:**
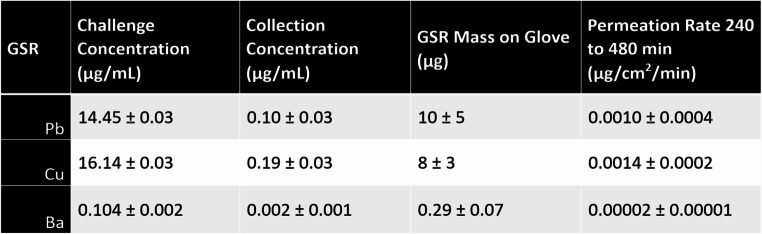
GSR parameters before and after permeation

For Pb, the original concentration in the GSR was 86.8 ± 0.2 mg/g, for Cu 96.4 ± 0.2 mg/g, and for Ba 0.622 ± 0.012 mg/g. To be noted were the respective metal glove contents of 10, 8.0 and 0.29 µg at 480 min that had been absorbed by the glove but not released into the collection fluid confirming metal transit through the glove. The method blank glove permeated ≤ 10 ng levels of these metals at 480 min.

That no metals permeated except in the 480 min sample implies the potential safe wearing resistance period of 240 min for this glove at these exposure conditions, a result described in the Kimtech Solvent Resistance Chart classification as High Protection [12] based on standardized breakthrough times in min of Not Recommended, < 10; Splash, 10–60; Medium Protection, 60–240; and High Protection, >240. The wearing time for most disposable gloves is short since they are designed for short-term exposure for example splash exposure, and are usually doffed before any work break for a maximum wearing time of 100–120 min. A place with hourly breaks would reduce this maximum wearing time even more. GSR of higher metal concentration than in the present study will permeate faster because of a higher concentration gradient in this kinetic system if the rest of the GSR chemical composition remains the same. The type of the metal compounds present is also important. Unburnt Pb styphnate (initiating explosive) and Ba nitrate (oxidizer) would be available to permeate [14]. Cu may be from the peening effect of the rifle chamber explosion during firing or as ultrathin bullet shavings, The factors that determine the GSR concentrations of these three elements are discussed more extensively elsewhere [15]. Many sampling and analytical techniques have been used to define the presence of these GSR elements in the environment [16, 17].

For people who perform the cleaning and maintenance of firearms, such as military personnel, law enforcement officers, gunsmiths, security personnel, hunters, sport marksmen, and other people who own or rent guns, there is likely a substantial risk of exposure to metallic GSRs when using GCSs if adequate gloves are not used as the primary dermal PPE of choice, based on the hand exposure data of Halim et al. 2013 [15] after a single firing of a pistol where a bare hand was exposed to 4–12 µg Pb, 1–9 µg Ba and 0.2–2.5 µg Cu.

Of these three elements Pb is of most concern since there is no known minimum adverse effect level for blood Pb relative to neurotoxic effects on young children and the newborn [18], followed by Ba [19] and Cu [20], the latter also being a human essential element. The low concentration of Ba also supports Pb as the primary toxicant at these challenge conditions. Therefore, the major populations at risk are the young children, neonates and fetuses of women of child-bearing age exposed to GSR such as female forensics and laboratory personnel, and those living near shooting ranges and near or on military grounds where ammunition is being tested or fired. An increase in challenge concentration of these elements will cause more to permeate and shorten the permeation detection breakthrough time. Other metals may also then permeate. Using Pb-free initiating explosive will also lower Pb exposure. Since most firers of loaded guns do not wear gloves, they may be exposed to much higher masses of GSR metals as demonstrated by Halim et al. 2013 [15].

It is hoped that the method development reported here will support future investigations and the generation of future data on protective glove permeation resistance and performance.

### Limitations

The present study focused just on the thickest disposable nitrile glove, Blue, of one major glove company, Kimtech Science, a division of Kimberly-Clark, and one type of GCS, gun and ammunition. Thinner disposable nitrile gloves and gloves other than nitrile may also show enough resistance to metallic compounds. Since GSR also has organic components, GSR permeation research should also be explored with other GCS, different glove materials, and ammunition to identify appropriate glove choices that do not obstruct gun firing, and to understand the risk for exposure to metal and organic GSRs during the cleaning and maintenance of firearms. This might produce enough data to develop regulations for skin exposure that currently do not exist.

## Conclusions

Three metal GSRs, Pb, Cu and Ba, can permeate through this disposable nitrile glove at these challenge conditions. However, the data also show that if this glove was doffed before 240 min the wearer should not be exposed to GSR metals during GSR removal with a popular GCS. For people who perform the cleaning and maintenance of firearms, such as military personnel, law enforcement officers, gunsmiths, security personnel, hunters, sport marksmen, and other people who own or rent guns, there is likely a substantial risk of exposure to metallic GSRs if adequate gloves are not used as the primary dermal PPE of choice during removal of GSR with GCSs.

### Key points

1. Assumptions about the protectiveness of personal protective equipment like gloves in the forensics and other sciences as well as in other situations where exposures to chemicals occur need to be proven to assure minimal toxic effects.
2. The elements in GSR dissolved in Hoppe’s number 9 gun bore cleaner that permeated a thick disposable nitrile glove did so between 240 min and 480 min in a modified ASTM F739 protocol.
3. The three elements that permeated were Pb, Cu and Ba that were also determined within the glove at higher levels relative to the glove blank.
4. The GSR concentrations in mg/g generated from firing 1000 rounds over 120 min were Pb 86.8 ± 0.2, Cu 96.4 ± 0.2, and Ba 0.622 ± 0.012.

## Declarations

### Rights and permissions

Open Access. The datasets generated during and/or analysed during the current study are available from the corresponding author on reasonable request.

## References

[1] Cribbs TD, Que Hee SS. Permeation of a firearm cleaning solvent through disposable nitrile gloves. J Occup Environ Hyg. 2024;21(7):494–03. 10.1080/15459624.2024.2345815. 38838303 10.1080/15459624.2024.2345815 PMC11479594

[2] Banaee S, Que Hee SS. Glove permeation of chemicals: The state of the art of current practice, Part 1: Basics and the permeation standards. J Occup Environ Hyg. 2019;12:827–39. 10.1080/15459624.2019.1678750. 10.1080/15459624.2019.1678754 PMC8005623 31684851

[3] Banaee S, Que Hee SS. Glove permeation of chemicals: The state of the art of current practice—Part 2. Research emphases on high boiling point compounds and simulating the donned glove environment. J Occup Environ Hyg. 2020;17(4):135–64. 10.1080/15459624.2020.1721509. 32209007 10.1080/15459624.2020.1721509 PMC7960877

[4] Kaur J, Sodhi GS. Forensic importance of gunshot residue analysis: A review. Int J Med Toxicol Legal Med. 2022;25(1–2):14–21. 10.5958/0974-4614.2022.00004.

[5] Minzière VR, Gassner A-L, Gallidabino M, Roux C, Weyermann C. The relevance of gunshot residues in forensic science. WIREs Forensic Sci. 2023;5:e1472. 10.1002/wfs2.1472.

[6] Gorey B, Boyle M, O’Brien CM, O’Shaughnessy J, Daly D, Forde A. Gunshot residue (GSR): frequency of residue types encountered in case work and background levels on control samples. Forensic Sci Int. 2024;359:112029. 10.1016/j.forsciint.2024.112029. 38657323 10.1016/j.forsciint.2024.112029

[7] Donghi M, Orsenigo S, Manna L, Profumo A, Mattino A, Merli D. Pervasiveness of inorganic gunshot residue (IGSR) in handguns after cleaning and conditioning procedures. J Forensic Sci. 2024;69(3):1035–44. 10.1111/1556-4029.15484. 38332695 10.1111/1556-4029.15484

[8] Burnett BR, Nunziata F. Divergent gunshot residues and characterization of the memory effect in a .22 caliber revolver and pistol. Egypt J Forensic Sci. 2023;13:13. 10.1186/s41935-023-00326-5

[9] Bushnell Holdings. Safety data sheet: Hoppe’s No. 9 Gun Bore Cleaner. 2019.

[10] ASTM International. ASTM F739-20. Standard test method for permeation of liquids and gases through protective clothing materials under conditions of continuous contact. West Conshohocken (PA). 2020.

[11] ASTM International. D6978 – 05 (Reapproved 2019): Standard practice for assessment of resistance of medical gloves to permeation by chemotherapy drugs. West Conshohocken (PA), 2019.

[12] Kimtech. Chemical Permeation Table 2025. Retrieved from https://www.kimtech.eu/resources/chemical-permeation/.

[13] Georgoulis LB, Morgan MS, Andrianopoulos N, Seferis JC. Swelling of polymeric glove materials during permeation by solvent mixtures. J Appl Polym Sci. 2005;97(3):775–83. 10.1002/app.21515.

[14] National Center for Forensic Science. 2025. https://ncfs.ucf.edu/research/chemical-evidence/gun-shot-residue/

[15] Halim MIA, Safian MF, Elias E, Shazali SS. Identification of gunshot residue from trace element by using icp/oes. 2013; IEEE Symposium on Computers & Informatics. pp 231-235. 10.1109/ISCI.2013.6612409

[16] Dalby O, Butler D, Birkett JW. Analysis of gunshot residue and associated materials –A review. J Forensic Sci. 2010;55(4):924–43. 20384934 10.1111/j.1556-4029.2010.01370.x

[17] Gong SA, Homberger N, Ling H. Elemental profiling of total gunshot residue using total reflection X-ray fluorescence spectrometry. J Forensic Sci. 2022;67(3):1198–207. 10.1111/1556-4029.14988. 35072267 10.1111/1556-4029.14988

[18] Agency for Toxic Substances and Disease Registry (ATSDR). Toxicological profile for lead. 2020. https://www.atsdr.cdc.gov/toxprofiles/tp13.pdf. 36888732

[19] Agency for Toxic Substances and Disease Registry (ATSDR). Toxicological profile for barium and barium compounds. 2007. https://www.atsdr.cdc.gov/toxprofiles/tp24.pdf. 38147518

[20] Agency for Toxic Substances and Disease Registry (ATSDR). Toxicological profile for copper. 2024. https://www.atsdr.cdc.gov/toxprofiles/tp132.pdf. 39689222

